# Increasing trends of overweight and obesity in treatment-naive people living with HIV in Shenzhen from 2014 to 2020: an emerging health concern

**DOI:** 10.3389/fpubh.2023.1186838

**Published:** 2023-10-10

**Authors:** Tianze Li, Liqin Sun, Yun He, Yang Zhou, Liumei Xu, Fang Zhao, Dongsheng Hu, Hui Wang, Hongzhou Lu, Jiaye Liu

**Affiliations:** ^1^School of Public Health, Shenzhen University Medical School, Shenzhen, China; ^2^Department of Infectious Diseases, National Clinical Research Center for Infectious Diseases, Shenzhen Third People's Hospital, Shenzhen, China

**Keywords:** treatment-naïve, people living with HIV, overweight, obesity, consecutive cross-sectional study

## Abstract

**Background:**

With the early initiation of antiretroviral therapy (ART) in China, the demographics of treatment-naïve people living with HIV (PLWH) are moving closer to those of the general population, which is characterized by a gradual increase in metabolic indicators. However, the epidemic trends of overweight and obesity over the past decade in treatment-naïve PLWH ready to initiate ART have not yet been investigated.

**Methods:**

A cross-sectional study was conducted, including 12,135 consecutive treatment-naïve PLWH ready to initiate ART in Shenzhen, using data retrieved from the China National Free Antiretroviral Treatment Program database from 2014 to 2020. The chi-square test was used to examine the trends of overweight and obesity between age groups, and multivariate logistic regression was used to identify the association of overweight and obesity with hyperglycemia and dyslipidemia.

**Results:**

During the 7-year study period, 12,135 treatment-naïve PLWH ready to initiate ART were included, among whom 1,837 (15.1%) were overweight and 388 (3.2%) were obese. The prevalence of overweight rose from 11.4 to 17.3% (*Z* = −4.58, *P* for trend <0.01) and that of obesity from 2.0% to 4.2% (*Z* = −6.45, *P* for trend <0.01) from 2014 to 2020. The annual prevalence of overweight was the highest in the age group of participants >35 years compared to prevalence in other age groups during the period 2014–2020. Compared with those who were not overweight or obese, PLWH who were overweight or obese were more likely to have hyperglycemia (aOR 1.84, 95% CI: 1.37–2.49 for overweight; aOR 2.68, 95% CI: 1.62–4.44 for obesity), higher ALT level (aOR 2.70, 95% CI: 2.33–3.13 for overweight; aOR 3.85, 95% CI: 2.93–5.05 for obesity), higher TG levels (aOR 1.89, 95% CI 1.63–2.19 for overweight; aOR 2.56, 95% CI 1.97–3.32 for obesity), and lower HDL levels (aOR 1.67, 95% CI 1.44–1.95 for overweight; aOR 2.06, 95% CI 1.54–2.77 for obesity).

**Conclusion:**

The prevalence of overweight and obesity in treatment-naive PLWH increased steadily from 2014 to 2020 in Shenzhen. Overweight and obese in treatment-naive PLWH ready to initiate ART were associated with dyslipidemia and hyperglycemia. Public health authorities should take proactive steps to address these issues by implementing targeted screening, intervention programs including lifestyle modifications, and integrated healthcare services.

## Introduction

Since the implementation of the national free antiretroviral therapy (ART) program for HIV in 2003, the mortality of people living with HIV (PLWH) has been effectively reduced and the life expectancy of PLWH has also been prolonged (1, 2). HIV infection has been transformed from a serious fatal disease to a chronic, manageable infectious disease; consequently, non-AIDS-defining diseases (NADs) have become the main burden of disease affecting PLWH, and one of the most common NADs is cardiovascular disease (CVD) (3). Therefore, early identification of high-risk groups and risk factors for CVD in PLWH and timely intervention are important. However, overweight and obesity as important risk factors for CVD have not attracted as much attention as dyslipidemia.

With the promotion of early treatment policies, the CD4+ T cell counts of PLWH at ART initiation are increasing (4) and the proportion of PLWH with advanced infection and wasting syndrome has gradually decreased. However, it is not clear whether the long-term trends in body weight among treatment-naïve PLWH ready to initiate ART are gradually becoming closer to those of the general population during the present epidemic of overweight and obesity (5). Existing evidence shows that the relative risk of CVD is generally 1.5-fold to 2-fold higher in PLWH than in people without HIV (6). It is well-established that overweight and obesity have consistently been recognized as risk factors for chronic conditions, including cardiovascular diseases and metabolic disorders, both in the general population and among PLWH. Furthermore, in the subsequent stages of antiretroviral treatment among PLWH, the effects of medication can contribute to increased susceptibility to weight gain (7, 8). Therefore, gaining insights into the trends of overweight and obesity is crucial in terms of clinical and public health implications. This understanding informs the selection of ART regimens for ARV-(antiretroviral)-naïve individuals, helps in reducing the risk of NADs, and benefits those PLWH who are on stable ARV treatment.

In this study, we conducted a consecutive cross-sectional analysis aiming to describe the overall trends of obesity and overweight in ARV-naive PLWH and to explore the factors associated with overweight and obesity in PLWH based on the data retrieved from China's National Free Antiretroviral Treatment Program (NFATP) database in Shenzhen from 2014 to 2020.

## Methods

### Study design and data source

We conducted a consecutive cross-sectional study of treatment-naive PLWH who sought care at the Third People's Hospital of Shenzhen during the period 2014–2020. All the data were retrieved from China's National Free Antiretroviral Treatment Program (NFATP) database. The NFATP is a community-based public health program and all PLWH enrolled in the free ART program receive long-term follow-up in China (9, 10). In this study, treatment-naïve PLWH ready to initiate ART were excluded if they were ≤ 15 years of age or they did not have a body mass index (BMI) measurement recorded in the database from 1 January 2014 to 31 December 2020. This study was approved by the Research Ethics Committee of the Third People's Hospital of Shenzhen (No. 2022-143), and all enrolled PLWH signed an informed consent form.

### Procedure

The outcomes of interest were overweight and obesity. According to the standards formulated by the China Obesity Working Group, a BMI of 24.0–27.9 kg/m^2^ was defined as overweight and a BMI of ≥28.0 kg/m^2^ was defined as obesity (11). We estimated the annual prevalence of overweight and obesity during 2014–2020. We described and compared the annual prevalence of overweight and obesity in different groups that were stratified by age (15–24, 25–34, ≥35 years), sex, and marital status. Groupings of baseline CD4+ T cell counts ( ≤ 199, 200–349, 350–499, ≥500 cells/μL) were based on the standard threshold for ART initiation in different study periods in China. Fasting blood glucose (Glu) exceeding 7 mmol/L was considered as hyperglycemia (12). Elevated levels of serum alanine aminotransferase (ALT) and aspartate aminotransferase (AST) were defined as levels >40 IU/L (13). According to the Chinese guidelines for the prevention and treatment of dyslipidemia in adults (14), triglyceride (TG) >2.26 mmol/L was defined as hypertriglyceridemia and total cholesterol (TC) >5.72 mmol/L was defined as hypercholesterolemia. Low levels of high-density lipoprotein cholesterol (HDL-C) and elevated low-density lipoprotein cholesterol (LDL) were defined as levels <0.91 mmol/L and >3.12 mmol/L, respectively. The intervals between HIV diagnosis and ART initiation were recorded.

### Statistical analysis

Categorical variables were described according to prevalence (%), and quantitative variables were described with median and interquartile range (IQR). The chi-square test was used for comparisons between categorical variables. We calculated the annual prevalence of overweight and obesity, further stratified by demographic and clinical factors, throughout the 7 years of the study. The factors of interest included age, sex, CD4+ T cell count, HDL-C, LDL-C, TC, TG, glucose, AST, ALT, WHO disease stage, marital status, plasma HIV RNA load, and interval between HIV diagnosis and ART initiation. Multivariate logistic regression modeling was used to calculate odd ratios (ORs) and 95% confidence intervals (CIs) for overweight and obesity. Results were considered statistically significant in the case of a *P*-value <0.05. All analyses were conducted with the use of SAS (version 9.1, SAS Institute, Cary, NC).

## Results

### The overall prevalence of overweight and obesity during the period 2014–2020

**Figure 1:**
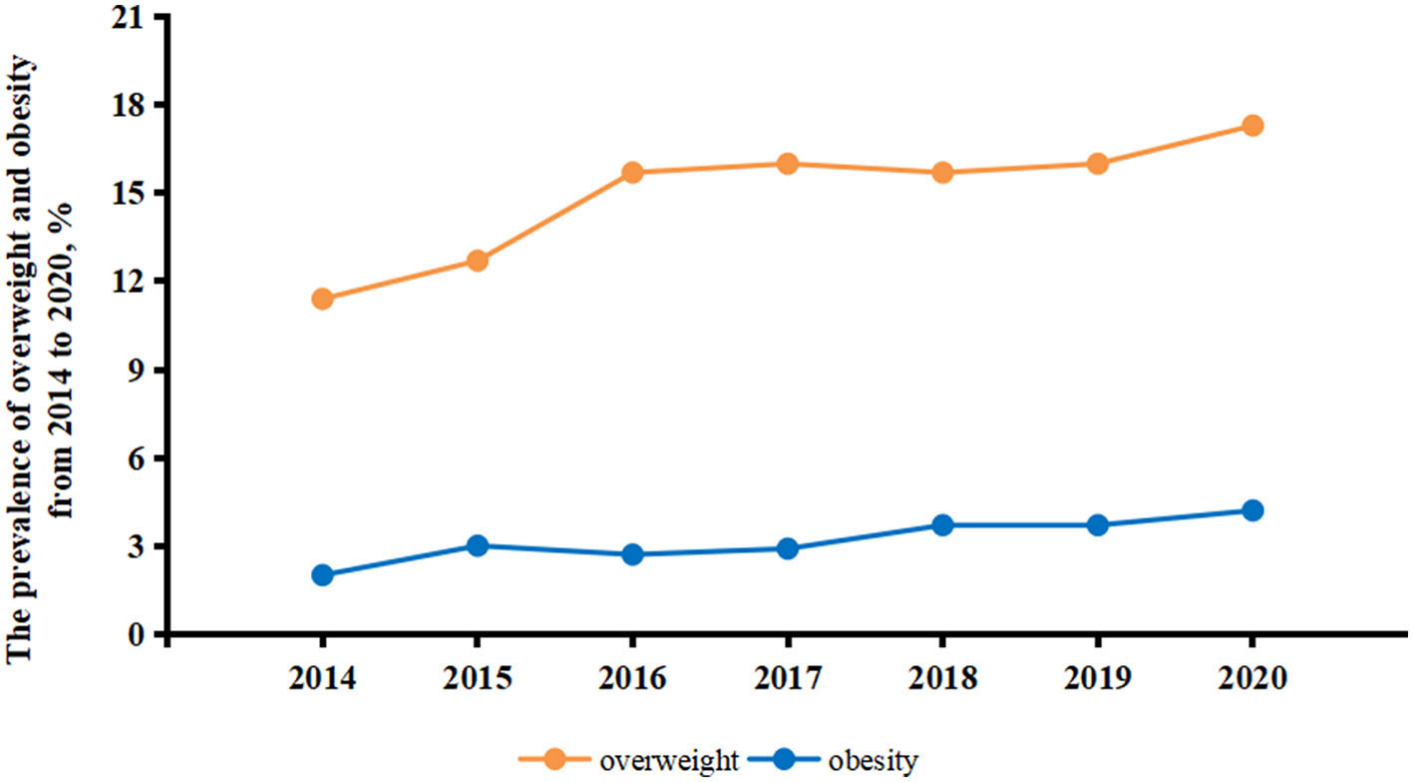
Prevalence of overweight and obesity in treatment-naïve PLWH ready to initiate ART from 2014 to 2020. Orange lines indicate the prevalence of overweight in treatment-naïve PLWH ready to initiate ART. Blue lines indicate the prevalence of obesity in treatment-naïve PLWH ready to initiate ART. ART, antiretroviral therapy; PLWH, people living with HIV.

A total of 12,135 treatment-naïve PLWH ready to initiate ART (11,102 men and 1,033 women) were included in this consecutive cross-sectional study. The median age of PLWH was 31 years (IQR 26–39), and the median CD4+ T cell count was 265 (IQR 171–366). The participants' main characteristics by year of enrollment are described in Table 1. Overall, 1,837 participants (15.1%) were overweight and 388 (3.2%) were obese. The prevalence of obesity and overweight generally showed a steadily increasing trend over the 7 years of the study, with the prevalence of overweight increasing from 11.4% in 2014 to 17.3% in 2020 (*Z* = −4.58, P for trend <0.01) and the prevalence of obesity increasing from 2.0% in 2014 to 4.2% in 2020 (*Z* = −6.45, P for trend <0.01) (Figure 1).

**Table 1:** Demographic and clinical characteristics of treatment-naïve PLWH with overweight and obesity during 2014–2020.

**Characteristic**	**2014**		**2015**		**2016**		**2017**		**2018**		**2019**		**2020**		**P1**	**P2**
		**(*N =* 948)**		**(*N =* 2,055)**		**(*N =* 2,150)**		**(*N =* 2,063)**		**(*N =* 1,867)**		**(*N =* 1,639)**		**(*N =* 1,413)**			
	**Overweight**	**Obesity**	**Overweight**	**Obesity**	**Overweight**	**Obesity**	**Overweight**	**Obesity**	**Overweight**	**Obesity**	**Overweight**	**Obesity**	**Overweight**	**Obesity**		
*n* (%)^a^	108 (11.4)	19 (2.0)	262 (12.7)	62 (3.0)	338 (15.7)	58 (2.7)	330 (16.0)	60 (2.9)	293 (15.7)	69 (3.7)	262 (16.0)	60 (3.7)	244 (17.3)	60 (4.2)	<0.01	<0.01
Age at start ART (years)	37.5 (31.0–44.0)	32.0 (28.0–43.0)	35.0 (28.0–42.0)	33.0 (28.0–38.0)	35.0 (28.0–43.0)	30.5 (28.0–38.0)	34.0 (28.0–42.0)	32 (28.5–41.0)	35.0 (29.0–43.0)	34.0 (28.0–41.0)	35.0 (28.0–44.0)	30.5 (27.0–37.0)	35.0 (28.0–45.0)	35.5 (27.5–42.0)	0.35	0.41
Sex															0.33	0.51
Men	97 (11.4)	18 (2.1)	249 (13.3)	54 (2.9)	319 (16.1)	56 (2.8)	305 (16.1)	57 (3.0)	278 (16.3)	65 (3.8)	245 (16.3)	55 (3.7)	223 (17.2)	57 (4.4)		
Women	11 (11.0)	1 (1.0)	13 (7.4)	8 (4.5)	19 (11.4)	2 (1.2)	24 (13.9)	3 (1.7)	15 (9.3)	4 (2.5)	17 (12.2)	5 (3.6)	21 (17.8)	3 (2.5)		
CD4+ (cells/μL)	259 (188.5–382)	289 (198–395)	293 (203–361)	284 (205–386)	307 (203–407)	354.5 (265–524)	299 (199–393)	305 (233.5–446)	287 (202–406)	326 (236–445)	304.5 (216–394)	353.5 (210.5–530)	322 (206.0–419.0)	308 (189–480)	0.35	0.09
<199	32 (9.1)	5 (1.4)	63 (10.3)	15 (2.5)	84 (13.6)	7 (1.1)	82 (13.1)	13 (2.1)	73 (11.6)	10 (1.6)	54 (10.0)	14 (2.6)	54 (13.0)	19 (4.6)		
200–349	40 (10)	8 (2.0)	119 (13.3)	28 (3.1)	122 (14.4)	21 (2.5)	132 (15.8)	22 (2.6)	108 (15.7)	31 (4.5)	104 (16.9)	16 (2.6)	90 (17.0)	16 (3.0)		
350–499	27 (17.8)	4 (2.6)	61 (14.0)	15 (3.4)	96 (19.2)	14 (2.8)	73 (19.7)	15 (4.1)	72 (19.2)	14 (3.7)	74 (22.0)	12 (3.6)	71 (22.0)	12 (3.7)		
≥500	9 (20.5)	2 (4.5)	19 (16.8)	4 (3.5)	36 (19.3)	16 (8.6)	42 (18.0)	10 (4.3)	40 (23.0)	14 (8.0)	30 (20.3)	18 (12.2)	29 (19.7)	13 (8.8)		
HDL (mmol/L)	1.09 (0.95–1.31)	1.11 (0.90–1.39)	1.26 (1.10–1.43)	1.28 (1.10–1.40)	1.28 (1.14–1.45)	1.24 (1.11–1.38)	1.10 (0.94–1.27)	1.07 (0.92–1.21)	1.07 (0.93–1.23)	1.08 (0.93–1.23)	1.12 (0.94–1.31)	1.05 (0.95–1.19)	1.04 (0.87–1.21)	1.02 (0.84–1.16)	<0.01	<0.01
<0.91	20 (18.7)	5 (4.7)	8 (10.3)	2 (2.6)	14 (12.6)	3 (2.7)	61 (19.1)	14 (4.4)	63 (19.5)	14 (4.8)	45 (18.0)	12 (4.8)	71 (22.4)	22 (6.9)		
≥0.91	88 (10.5)	14 (1.7)	254 (12.8)	60 (3.0)	324 (15.9)	55 (2.7)	268 (15.4)	46 (2.6)	230 (14.9)	55 (3.6)	217 (15.6)	48 (3.5)	173 (15.8)	38 (3.5)		
LDL (mmol/L)	2.48 (2.02–2.95)	2.56 (2.21–3.07)	2.62 (2.23–3.08)	2.84 (2.51–3.42)	2.79 (2.40–3.26)	2.89 (2.40–3.35)	2.64 (2.28–3.11)	2.81 (2.21–3.29)	2.58 (2.14–3.04)	2.76 (2.31–3.17)	2.58 (2.23–3.05)	2.69 (2.39–3.15)	2.71 (2.23–3.18)	2.68 (2.20–3.19)	<0.01	0.37
≤ 3.12	88 (10.7)	15 (1.8)	198 (11.8)	37 (2.2)	237 (14.0)	35 (2.1)	247 (14.5)	42 (2.5)	228 (14.4)	49 (3.1)	201 (14.6)	44 (3.2)	181 (15.6)	44 (3.8)		
>3.12	20 (15.6)	4 (3.1)	64 (17.0)	25 (6.6)	101 (22.2)	23 (5.1)	82 (23.2)	18 (5.1)	65 (22.8)	20 (7.0)	61 (23.3)	16 (6.1)	63 (24.9)	16 (6.3)		
TC (mmol/L)	4.44 (3.84–5.08)	4.60 (3.99–5.10)	4.32 (3.89–4.87)	4.06 (3.81–4.51)	4.29 (3.79–4.89)	4.38 (3.85–4.97)	4.37 (3.94–4.96)	4.51 (3.81–5.01)	4.29 (3.75–4.86)	4.64 (4.04–5.21)	4.22 (3.70–4.87)	4.31 (3.85–5.04)	4.23 (3.65–4.93)	4.33 (3.72–5.17)	0.15	0.4
≤ 5.72	106 (11.4)	17 (1.8)	249 (12.4)	60 (3.0)	328 (15.6)	55 (2.6)	317 (15.8)	57 (2.8)	282 (15.4)	68 (3.7)	256 (15.9)	59 (3.7)	235 (16.9)	57 (4.1)		
>5.72	2 (9.1)	2 (9.1)	13 (25.5)	2 (3.9)	10 (21.3)	3 (6.4)	12 (21.8)	3 (5.5)	11 (32.4)	1 (2.9)	6 (20.7)	1 (3.4)	9 (20.7)	3 (3.4)		
TG (mmol/L)	1.66 (1.25–2.33)	2.35 (1.61–4.30)	1.65 (1.15–2.31)	1.90 (1.30–2.31)	1.41 (1.05–2.04)	2.09 (1.24–2.87)	1.44 (1.12–2.08)	1.59 (1.21–2.44)	1.41 (1.00–1.92)	1.49 (1.07–2.19)	1.34 (0.93–1.80)	1.35 (1.00–1.85)	1.48 (1.13–2.05)	1.75 (1.33–2.59)	<0.01	<0.01
<2.26	74 (9.4)	9 (1.1)	194 (11.1)	43 (2.5)	269 (14.5)	37 (2.0)	257 (14.1)	42 (2.3)	244 (14.4)	53 (3.1)	226 (15.0)	50 (3.3)	193 (15.5)	42 (3.4)		
≥2.26	34 (21.3)	10 (6.3)	68 (22.7)	19 (6.3)	69 (23.4)	21 (7.1)	72 (29.8)	18 (7.4)	49 (29.2)	16 (9.5)	36 (27.5)	10 (7.6)	51 (30.0)	18 (10.6)		
Glu (mmol/L)	5.16 (4.81–5.43)	5.18 (4.83–5.56)	5.17 (4.85–5.45)	5.28 (4.81–5.77)	5.23 (4.92–5.61)	5.26 (4.96–5.55)	5.26 (4.95–5.59)	5.31 (4.96–5.89)	5.19 (4.89–5.65)	5.30 (4.88–5.62)	5.30 (4.98–5.58)	5.34 (4.93–5.81)	5.18 (4.79–5.61)	5.27 (4.95–5.79)	0.02	0.88
<7.0	105 (11.5)	19 (2.3)	250 (12.8)	57 (3.2)	321 (15.6)	54 (3.0)	316 (16.1)	53 (3.1)	279 (15.9)	65 (4.2)	251 (16.3)	58 (4.3)	232 (17.5)	57 (5.0)		
≥7.0	3 (20.0)	0 (0)	12 (33.3)	5 (17.2)	17 (51.5)	4 (20.0)	13 (31.0)	7 (19.4)	14 (35.0)	4 (19.4)	11 (26.8)	2 (6.3)	12 (42.9)	3 (15.8)		
WHO disease stage															0.03	0.21
1	24 (17.1)	6 (4.3)	74 (14.4)	21 (4.1)	128 (19.3)	28 (4.2)	109 (19.2)	25 (4.4)	104 (20.2)	29 (5.6)	102 (22.8)	28 (6.3)	98 (20.1)	26 (5.3)		
2	49 (12.2)	8 (2.0)	123 (13.9)	25 (2.8)	122 (15.0)	20 (2.5)	134 (16.8)	21 (2.6)	104 (16.2)	28 (4.4)	99 (17.1)	15 (2.6)	83 (18.4)	14 (3.1)		
3	28 (10.4)	5 (1.9)	59 (12.3)	13 (2.7)	75 (16.3)	8 (1.7)	69 (14.8)	10 (2.1)	71 (14.9)	7 (1.5)	52 (11.8)	16 (3.6)	49 (15.4)	14 (4.4)		
4	7 (5.1)	0 (0)	6 (3.4)	3 (1.7)	13 (6.0)	2 (0.9)	17 (7.4)	4 (1.7)	14 (6.0)	5 (2.1)	9 (5.1)	1 (0.6)	14 (9.0)	6 (3.8)		
AST (U/L)	23.0 (19.0–30.5)	21.0 (17.0–29.0)	23.0 (19.0–29.0)	26.0 (22.0–36.0)	23.0 (19.0–29.0)	26.5 (20.0–41.0)	24.0 (20.0–32.0)	27.0 (20.5–44.5)	26.0 (21.0–34.0)	29.0 (23.0–48.0)	25.0 (21.0–31.0)	30.0 (24.0–44.5)	24.0 (20.0–32.0)	28.5 (2.5–46.0)	<0.01	0.02
≤ 40	106 (11.4)	19 (2.0)	257 (12.7)	60 (3.0)	335 (16.0)	54 (2.6)	320 (16.0)	56 (2.8)	284 (15.8)	65 (3.6)	252 (16.0)	57 (3.6)	238 (16.0)	56 (3.6)		
>40	2 (10.5)	0 (0.0)	5 (13.5)	2 (5.4)	3 (5.7)	4 (7.5)	9 (14.5)	4 (6.5)	9 (13.6)	4 (6.1)	10 (15.9)	3 (4.8)	6 (15.0)	4 (10.0)		
ALT (U/L)	19.0 (13.0–29.2)	18.0 (13.0–28.5)	20.0 (14.0–30.0)	19.0 (14.0–29.0)	20.0 (15.0–30.0)	20.0 (14.0–30.0)	22.0 (6.0–34.0)	22.0 (15.0–33.0)	16.0 (12.0–23.0)	23.0 (16.0–35.0)	21.0 (15.0–33.0)	21.0 (15.0–32.0)	20.0 (14.0–33.0)	19.0 (13.0–32.0)	0.43	<0.01
≤ 40	105 (11.5)	19 (2.1)	248 (12.5)	54 (2.7)	321 (15.6)	47 (2.3)	302 (15.5)	45 (2.3)	262 (15.1)	53 (3.0)	246 (15.8)	43 (2.8)	227 (17.0)	45 (3.4)		
>40	3 (8.6)	0 (0.0)	14 (21.2)	8 (12.1)	17 (18.9)	11 (12.2)	27 (24.3)	15 (13.5)	31 (24.4)	16 (12.6)	16 (18.6)	17 (19.8)	17 (22.7)	15 (20.0)		
Marital status															<0.01	0.25
Single	35 (6.7)	7 (1.3)	130 (10.7)	29 (2.4)	162 (12.2)	34 (2.6)	170 (13.2)	33 (2.6)	136 (11.9)	36 (3.2)	128 (12.6)	37 (3.6)	77 (14.8)	22 (4.2)		
Married	61 (18.3)	11 (3.3)	96 (15.7)	23 (3.8)	141 (23.1)	19 (3.1)	125 (20.6)	21 (3.5)	116 (21.0)	23 (4.2)	90 (19.8)	17 (3.7)	37 (18.3)	9 (4.5)		
Divorced	11 (13.3)	1 (1.2)	36 (17.5)	10 (4.9)	33 (18.6)	5 (2.8)	30 (20.7)	4 (2.8)	40 (25.2)	9 (5.7)	42 (27.5)	6 (3.9)	20 (27.0)	9 (12.2)		
Widowed	1 (10.0)	0 (0.0)	0 (0.0)	0 (0.0)	2 (6.1)	0 (0.0)	4 (16.0)	2 (8.0)	1 (6.3)	1 (6.3)	2 (12.5)	0 (0.0)	1 (16.7)	0 (0.0)		
HIV RNA (copies/mL)															<0.01	<0.01
<100000	60 (11.9)	8 (1.6)	147 (13.3)	28 (2.5)	126 (16.1)	20 (2.6)	183 (16.8)	33 (3.0)	152 (16.1)	43 (4.6)	146 (16.4)	40 (4.5)	123 (17.4)	36 (5.1)		
100000–999999	37 (10.4)	7 (2.0)	90 (11.6)	31 (4.0)	140 (16.1)	20 (2.3)	122 (15.0)	24 (3.0)	123 (16.2)	23 (3.0)	98 (15.9)	18 (2.9)	95 (17.0)	22 (3.9)		
>1000000	11 (12.9)	4 (4.5)	25 (14.4)	3 (1.7)	72 (15.0)	18 (3.6)	24 (14.8)	3 (1.9)	18 (11.3)	3 (1.8)	18 (13.5)	2 (1.5)	26 (17.8)	2 (1.4)		
Time Interval (Months)																
<12	79 (10.9)	14 (1.9)	197 (12.8)	39 (2.5)	270 (15.8)	39 (2.3)	291 (16.4)	46 (2.6)	251 (15.2)	60 (3.6)	237 (15.8)	53 (3.5)	223 (17.1)	50 (3.8)	<0.01	<0.01
≥12	29 (13.1)	5 (2.3)	65 (12.5)	23 (4.4)	68 (15.5)	19 (4.3)	38 (13.3)	14 (4.9)	42 (19.7)	9 (4.2)	25 (17.7)	7 (5.0)	21 (19.6)	10 (9.3)		

Data are presented as n (%), median (IQR). ALT, alanine aminotransferase; AST, aspartate transaminase; Glu, glucose; HDL, high-density lipoprotein; IQR, interquartile range; LDL, low-density lipoprotein; PLWH, people living with HIV; TC, total cholesterol; TG, triacylglycerol; WHO, World Health Organization. ^a^The number of people who were overweight or obese in the current year and their percentage of the total number of people in the current year.

### The trends of overweight and obesity in different groups from 2014 to 2020

The prevalence of overweight among men gradually increased from 11.7% in 2014 to 18.0% in 2020 (*Z* = –4.37, P for trend <0.01), and the prevalence of obesity increased from 2.4% in 2014 to 5.3% in 2020 (*Z* = –3.80, P for trend <0.01). However, similar trends were not observed in women (11.0% in 2014 vs. 17.8% in 2020 for overweight, *Z* = –1.95, P for trend = 0.05; 1.1% in 2014 vs. 3.1% in 2020 for obesity, *Z* = –3.97, P for trend = 0.69).

**Figure 2:**
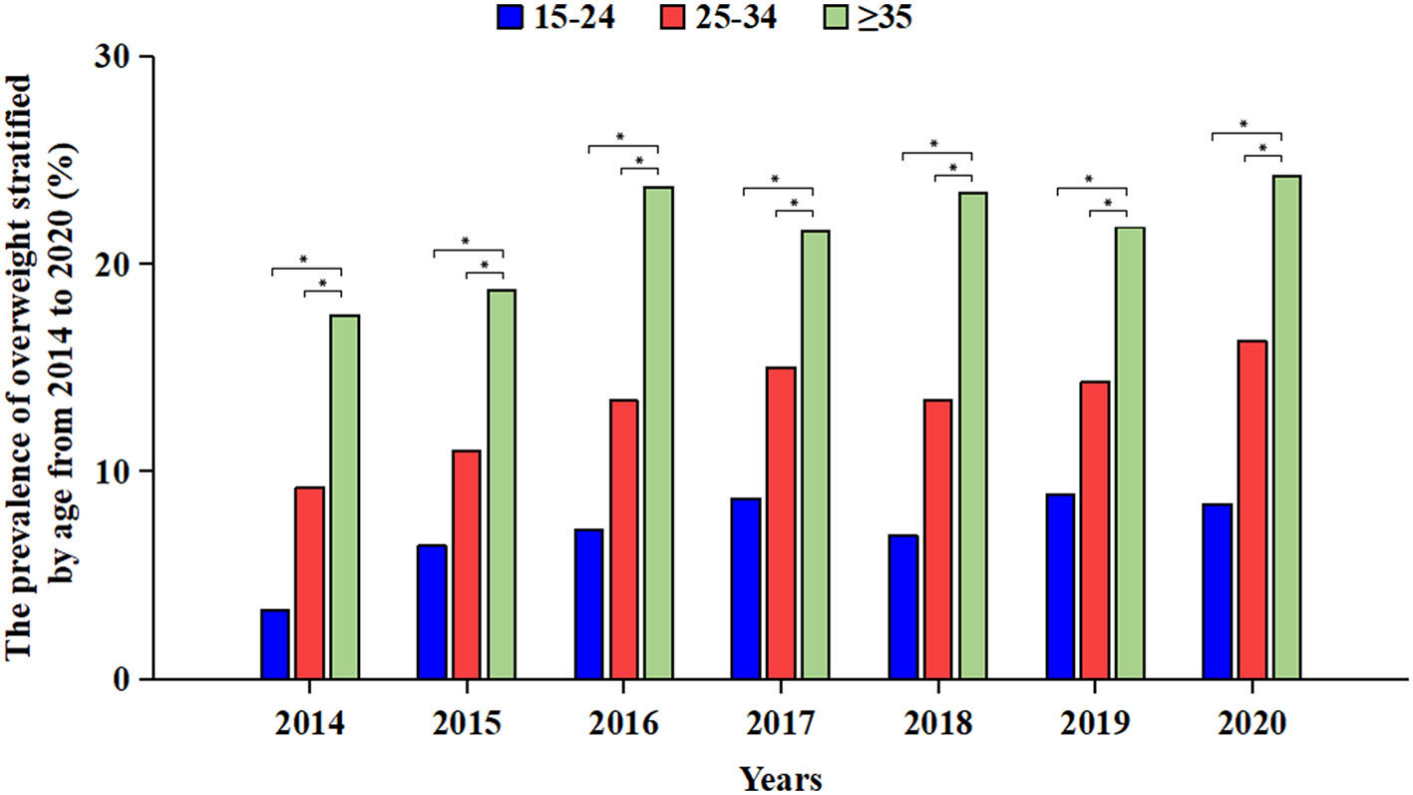
Prevalence of overweight in treatment-naïve PLWH ready to initiate ART stratified by age group from 2014 to 2020. Blue bars indicate the prevalence among 15–24-year-old treatment-naïve PLWH ready to initiate ART. Red bars indicate the prevalence among 25–34-year-old treatment-naïve PLWH ready to initiate ART. Green bars indicate the prevalence among ≥35-year-old treatment-naïve PLWH ready to initiate ART. *Indicates that there is a statistically significant difference between the two groups. ART, antiretroviral therapy; PLWH, people living with HIV.

We calculated the prevalence of overweight and obesity in different age groups from 2014 to 2020 to examine the trends of overweight and obesity with age in treatment-naive PLWH. The results showed that the prevalence of overweight and obesity in PWLH aged 15–24, 25–34, and ≥35 years increased consistently in all cases over the 7-year period. For overweight, the prevalence increased from 3.3% in 2014 to 8.4% in 2020 in the 15- to 24-year-old age group (χ^2^ = 6.87, *P* = 0.33). In the 25- to 34-year-old group, the prevalence increased from 9.2% in 2014 to 16.3% in 2020 (χ^2^ = 18.75, *P* < 0.01); in the ≥35-year-old group, the prevalence increased from 17.5% in 2014 to 24.2% in 2020 (χ^2^ = 18.33, *P* < 0.01). In each year, the prevalence of overweight remained the highest in the ≥35-year-old group, followed by the 25- to 34-year-old group, and the prevalence in the 15- to 24-year-old group remained the lowest (Figure 2).

**Figure 3:**
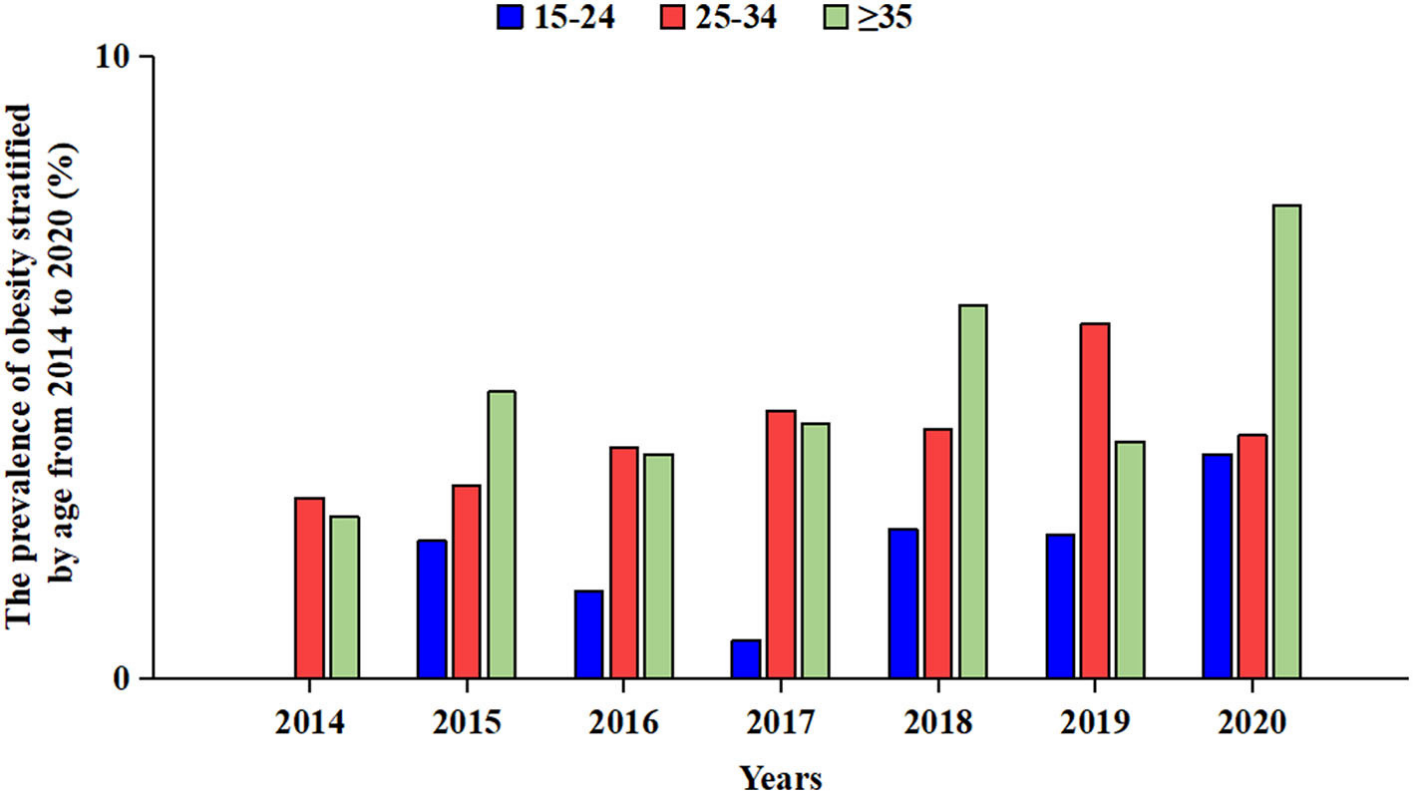
Prevalence of obesity in treatment-naïve PLWH ready to initiate ART stratified by age group from 2014 to 2020. Blue bars indicate the prevalence among 15–24-year-old treatment-naïve PLWH ready to initiate ART. Red bars indicate the prevalence among 25–34-year-old treatment-naïve PLWH ready to initiate ART. Green bars indicate the prevalence of ≥35-year-old treatment-naïve PLWH ready to initiate ART. ART, antiretroviral therapy; PLWH, people living with HIV.

For obesity, the prevalence of treatment-naïve PLWH ready to initiate ART in the 15- to 24-year-old group increased from 0 to 3.6% throughout the 7 years and the corresponding prevalence in the 25- to 34-year-old group increased from 2.9% to 3.9% (χ^2^ = 7.92, *P* = 0.24), while the corresponding prevalence of the ≥35-year-old group increased from 2.6% to 7.6% in 2014 to 2020 (χ^2^ = 15.94, *P* = 0.01). In each year, we found that the prevalence in the 15- to 24-year group remained lowest from 2014 to 2020, while we did not find a difference in the prevalence between the 25- to 34-year-old and ≥35-year-old groups (Figure 3).

### Association of overweight and obesity with metabolic abnormalities

Our results indicated that PLWH who were overweight or obese had a higher prevalence of metabolic abnormalities than those without overweight or obesity.

The overweight PLWH population had higher prevalence of abnormal LDL-C levels (24.8% for overweight vs. 15.5% for normal BMI, χ^2^ = 96.41, *P* < 0.01), HDL-C (15.7% for overweight vs. 11.7% for normal BMI, χ^2^ = 22.99, *P* < 0.01), TC (4.1% for overweight vs. 2.1% for normal BMI, χ^2^ = 28.83, *P* < 0.01), TG (20.6% for overweight vs. 9.8% for normal BMI, χ^2^ = 177.33, *P* < 0.01), AST (13.6% for overweight vs. 12.4% for normal BMI, χ^2^ = 2.15, *P* = 0.14), ALT (9.2% for overweight vs. 1.7% for normal BMI, χ^2^ = 48.98, *P* < 0.01), and glucose (4.4% for overweight vs. 1.6% for normal BMI, χ^2^ = 62.44, *P* < 0.01) than those of the PLWH population with normal BMI.

In the obese population, we observed a more pronounced increasing trend in the prevalence of abnormal metabolic indicators. The rate of abnormal LDL-C increased from 15.5% to 31.4% (χ^2^ = 70.52, *P* < 0.01), HDL-C increased from 11.7% to 19.1% (χ^2^ = 16.27, *P* < 0.01), AST increased from 12.4% to 14.9% (χ^2^ = 55.23, *P* < 0.01), and ALT increased from 7.1% to 25.3% (χ^2^ =276.76, *P* < 0.01). Abnormal TC rates increased by 2.3% (4.4% for obesity vs. 2.1% for normal BMI, χ^2^ = 70.52, *P* < 0.01), abnormal TG rates increased by 19.1% (28.9% for obesity vs. 9.8% for normal, χ^2^ = 143.19, *P* < 0.01), and abnormal glucose rates increased by 4.8% (6.4% for obesity vs. 1.6% for normal BMI, χ^2^ = 50.82, *P* < 0.01).

### Multivariate logistic regression analyses of factors associated with overweight and obesity

**Figure 4:**
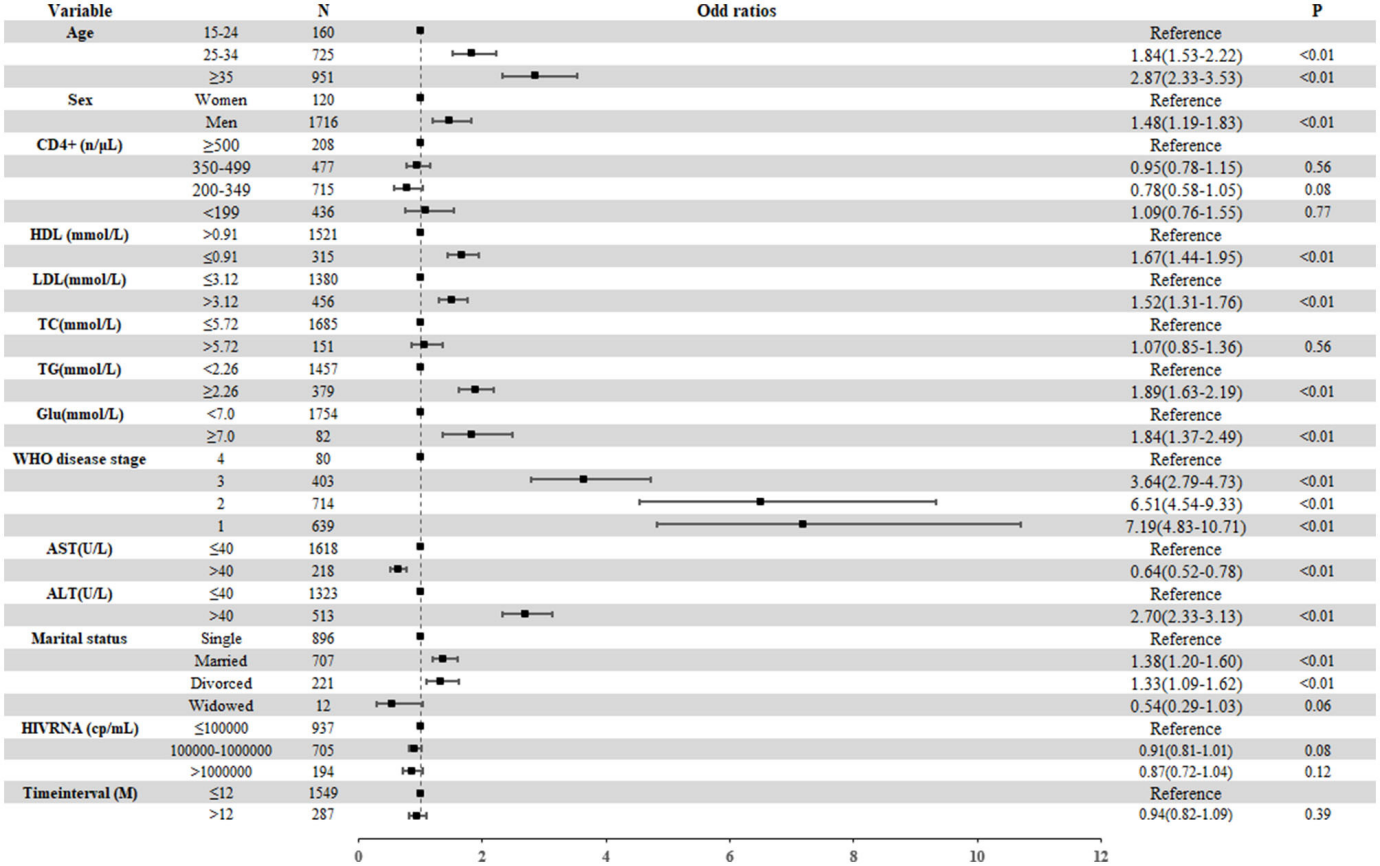
Factors related to the overweight among treatment-naïve PLWH ready to initiate ART from 2014 to 2020. The boxes represent the odds ratio, and the short horizontal lines represent 95% CIs. ALT, alanine aminotransferase; AST, aspartate transaminase; Glu, glucose; HDL, high-density lipoprotein; IQR, interquartile range; LDL, low-density lipoprotein; PLWH, people living with HIV; TC, total cholesterol; TG, triacylglycerol; WHO, World Health Organization.

The results of multivariate logistic regression analysis indicated that, compared with PLWH who had normal BMI, PLWH with overweight were more likely to be male (aOR 1.48, 95% CI 1.19–1.83) and to have lower HDL-C (aOR 1.67, 95% CI 1.44–1.95), higher LDL-C (aOR 1.52, 95% CI 1.31–1.76 for overweight), higher TC (aOR 1.89, 95% CI 1.63–2.19 for overweight), higher ALT levels (aOR 2.70, 95% CI 2.33–3.13), and hyperglycemia (aOR 1.84, 95% CI 1.37–2.49). In contrast, higher AST levels (aOR 0.64, 95% CI 0.52–0.78) were strongly statistically correlated with a lower risk of overweight. In addition, a significant statistical correlation between WHO disease stage and overweight was observed. The strength of the correlation decreased with progression of the disease stage. Compared with PLWH in stage 4, the aORs for stages 1–3 were 7.19 (95% CI 4.83–10.71), 6.51 (95% CI 4.54–9.33), and 3.64 (95% CI 2.79–4.73), respectively. We found no significant correlations between CD4+ T cell counts or plasma HIV RNA load and overweight (Figure 4).

**Figure 5:**
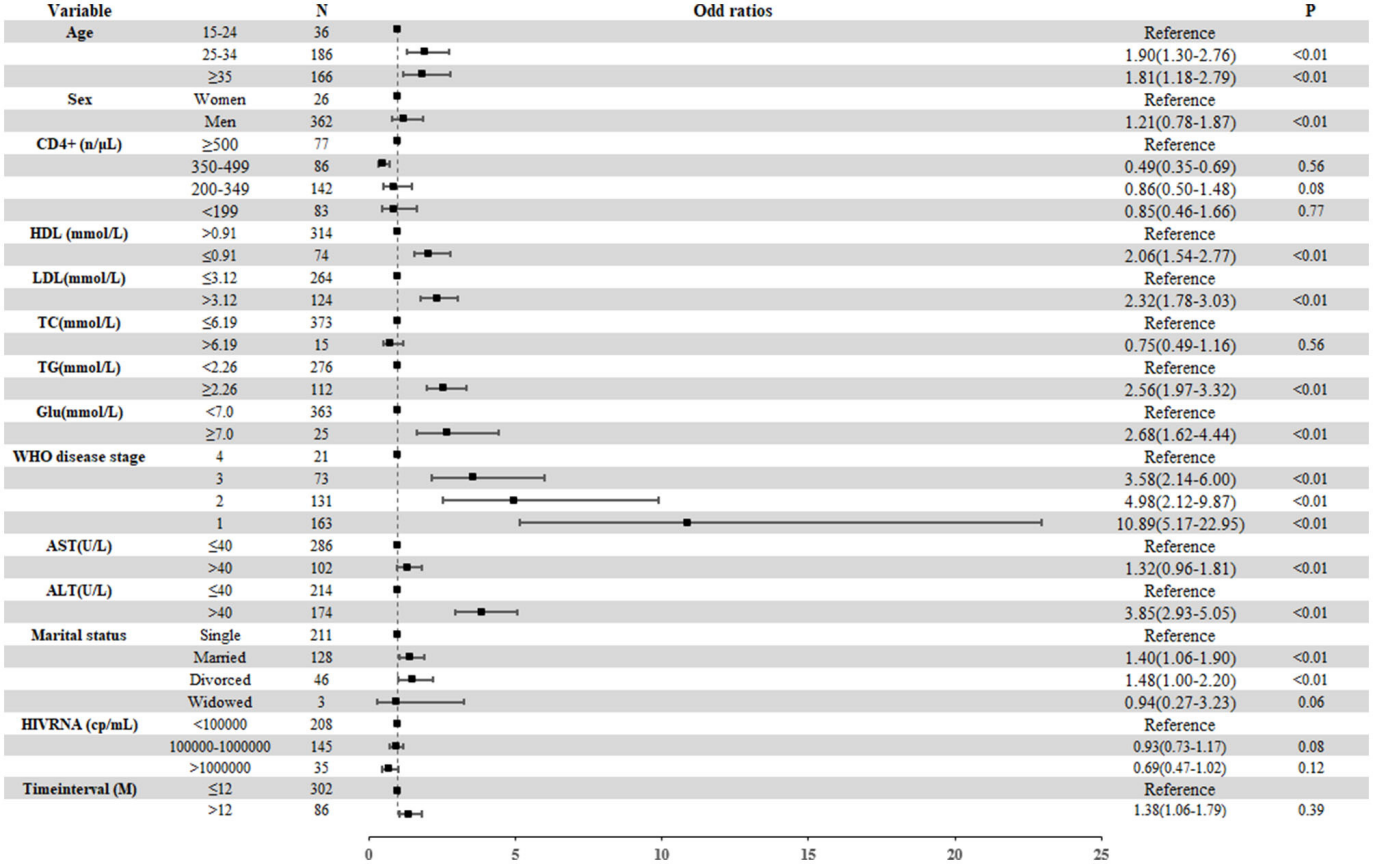
Factors related to the obesity among treatment-naïve PLWH ready to initiate ART from 2014 to 2020. The boxes represent the odds ratio, and the short horizontal lines represent 95% CIs. ALT, alanine aminotransferase; AST, aspartate transaminase; Glu, glucose; HDL, high-density lipoprotein; LDL, low-density lipoprotein; TC, total cholesterol; TG, triglyceride; WHO, World Health Organization.

For PLWH with obesity, the results of the multivariate logistic regression model were similar to those with overweight. Obese PLWH were more likely to have higher TG (aOR 2.56, 95% CI 1.97–3.32), higher ALT (aOR 3.85, 95% CI 2.93–5.05), lower HDL (aOR 2.06, 95% CI 1.54–2.77), and hyperglycemia (aOR 2.68, 95% CI 1.62–4.44). It seemed that age was not associated with obesity. Compared with PLWH in stage 4, progression to stage 1 (aOR 10.89, 95% CI 5.17–22.95), stage 2 (aOR 4.98, 95% CI 2.12–9.87), and stage 3 (aOR 3.58, 95% CI 2.14–6.00) also had a stepwise decreasing correlation with obesity (Figure 5). Finally, we did not find a significant correlation of sex, CD4+ T cell counts, TC levels, AST levels, or HIV RNA viral load with obesity.

## Discussion

In this surveillance study, we examined the trends in overweight and obesity and identified factors associated with these conditions among treatment-naive PLWH in China from 2014 to 2020. The present results demonstrated that the prevalence of overweight and obesity in treatment-naive PLWH continued increasing year by year from 2014 to 2020. Compared with 2014, the prevalence of overweight had increased by 54.4% and the prevalence of obesity increased by 110% by 2020. The association of overweight and obesity with dyslipidemia and hyperglycemia highlights the urgent need for interventions, given the increased risk of CVD for people with dyslipidemia and hyperglycemia.

This study found that the prevalence of overweight and obesity was steadily increasing among treatment-naïve PLWH ready to initiate ART over the past 7 years. Our results were consistent with the findings based on the Chinese Health and Nutrition Survey (15), which suggested that the BMIs of men and women in the general population were steadily increasing during the period 2015–2019. This implies that the risk of CVD in treatment-naïve PLWH ready to initiate ART has been increasing over the years. Moreover, with the early initiation of ART, regardless of the regimens chosen, weight gain will occur as a phenomenon of “return to health” in the first couple of years following ART initiation (16, 17), which is likely to worsen the trends of overweight and obesity in the long term. Our findings suggest that it is essential to strengthen the surveillance and management of weight at the initiation of ART and during long-term follow-up.

In our study, we found that PLWH who were overweight or obese were more likely to have metabolic abnormalities. Notably, metabolic abnormalities generally manifest as aggregated abnormalities in multiple indicators, including dyslipidemia and impaired glucose homeostasis. The management of these issues will become increasingly important as ART is initiated. HIV infection is characterized by persistent inflammation and immune activation (18), which has the effect of enhancing insulin resistance and accelerating the metabolic dysregulation of adipose tissue and the liver (19, 20). Evidence from numerous epidemiology studies indicates that the prevalence of hyperlipidemia among PLWH ranges from 28 to 80%. Hypertriglyceridemia is usually the most obvious abnormality and appears to be an independent factor in the increased cardiovascular risk in this population (21). Hypertriglyceridemia among HIV patients is associated with a higher intake of total fat, saturated fat, trans fat, and cholesterol compared to HIV-negative controls (22). Excessive energy intake is also associated with a higher risk of overweight and obesity. However, obesity in turn can lead to metabolic imbalance, which can create a vicious cycle in the body. Therefore, an optimal weight management program needs to take into consideration both overweight and other metabolic abnormalities.

Our results showed that PLWH who initiated ART at earlier WHO stages of HIV were more likely to be overweight and obese. As the disease progresses, CD4+ T cells will continue to be depleted and the inflammatory markers of the hypothalamus will increase. Treatment-naïve PLWH ready to initiate ART may have anorexia symptoms, resulting in lower energy intake (23). During infection, the basic metabolic demands of the body can exceed 30% of the baseline, and the increase in protein turnover will lead to a catabolic state (24, 25). The above two factors work together to reduce the prevalence of overweight and obesity in patients at an advanced WHO disease stage. With the scaling up of early ART, the proportion of patients with WHO stages 1 and 2 continues to increase, so we should pay more attention to the overweight and obese people in stages 1 and 2.

There are several limitations to our study. First, we only included treatment-naïve PLWH ready to initiate ART, and those who were not ready or not eligible to start ART, including undiagnosed and untreated PLWH, were not included. We might have underestimated the prevalence of overweight or obesity because those who were not eligible to start ART could be in the early stage of HIV infection and have higher CD4 counts. Second, the information recorded in the NFATP database is incomplete, with a lack of data on factors such as smoking, drinking, lifestyle, and family history; therefore, we were not able to examine the impact of these factors on the trends in overweight and obesity observed in this study. Finally, the cross-sectional study design precluded us from demonstrating a causal relationship between overweight and obesity and the associated factors identified.

## Conclusion

In conclusion, the prevalence of overweight and obesity in Chinese treatment-naive PLWH increased year by year from 2014 to 2020. The study reinforces that while metabolic abnormalities may not have an immediate and severe impact on the health of PLWH, they significantly increase the risk of developing chronic conditions like cardiovascular diseases in the future. To combat the rising prevalence of overweight and obesity in Chinese treatment-naïve PLWH, we suggest that public health professionals should focus on community-level interventions including health education programs to raise awareness and behavioral lifestyle interventions to promote healthier choices.

## Data availability statement

The raw data supporting the conclusions of this article will be made available by the authors, without undue reservation.

## Ethics statement

The studies involving humans were approved by the Research Ethics Committee of the Third People's Hospital of Shenzhen. The studies were conducted in accordance with the local legislation and institutional requirements. Written informed consent for participation in this study was provided by the participants' legal guardians/next of kin.

## Author contributions

TL and LS conceived the study, conducted the interview, performed the formal analysis, and interpreted the data. JL, HL, and HW supervised the study, obtained ethics approval, and had full access to all of the data in the study. JL and LS wrote the manuscript. All authors have critically read and approved the manuscript.
